# Technological advances and challenges in constructing complex gut organoid systems

**DOI:** 10.3389/fcell.2024.1432744

**Published:** 2024-08-14

**Authors:** Longjin Zheng, Yang Zhan, Chenxuan Wang, Qigui Fan, Denglong Sun, Yingmeng Li, Yanxia Xiong

**Affiliations:** ^1^ State Key Laboratory for the Modernization of Classical and Famous Prescriptions of Chinese Medicine, Nanchang, China; ^2^ Research and Development Department, Jiangzhong Pharmaceutical Co., Ltd., Nanchang, China

**Keywords:** gut organoid, 3D bioprinting, microfluidics, co-culture systems, organ-on-a-chip

## Abstract

Recent advancements in organoid technology have heralded a transformative era in biomedical research, characterized by the emergence of gut organoids that replicate the structural and functional complexity of the human intestines. These stem cell-derived structures provide a dynamic platform for investigating intestinal physiology, disease pathogenesis, and therapeutic interventions. This model outperforms traditional two-dimensional cell cultures in replicating cell interactions and tissue dynamics. Gut organoids represent a significant leap towards personalized medicine. They provide a predictive model for human drug responses, thereby minimizing reliance on animal models and paving the path for more ethical and relevant research approaches. However, the transition from basic organoid models to more sophisticated, biomimetic systems that encapsulate the gut’s multifaceted environment—including its interactions with microbial communities, immune cells, and neural networks—presents significant scientific challenges. This review concentrates on recent technological strides in overcoming these barriers, emphasizing innovative engineering approaches for integrating diverse cell types to replicate the gut’s immune and neural components. It also explores the application of advanced fabrication techniques, such as 3D bioprinting and microfluidics, to construct organoids that more accurately replicate human tissue architecture. They provide insights into the intricate workings of the human gut, fostering the development of targeted, effective treatments. These advancements hold promise in revolutionizing disease modeling and drug discovery. Future research directions aim at refining these models further, making them more accessible and scalable for wider applications in scientific inquiry and clinical practice, thus heralding a new era of personalized and predictive medicine.

## Graphical Abstract

**Figure:**
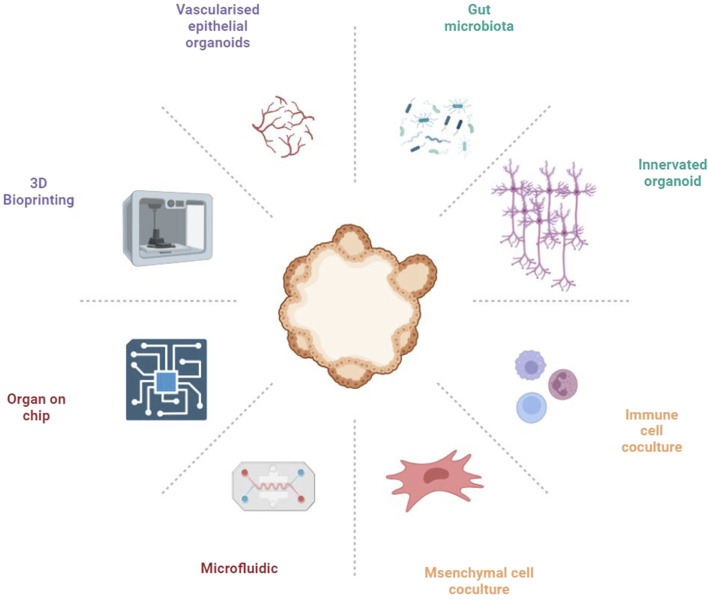

## 1 Introduction

Organoid technology signifies a paradigm shift in biomedical research, introducing a platform that mirrors the structural and functional complexity of human organs. This innovative approach has gained popularity due to its potential to revolutionize both scientific inquiry and clinical practice. Unlike traditional two-dimensional cell cultures that provide a limited perspective on cellular interactions and organ dynamics, organoids offer a three-dimensional environment where cells can self-organize into structures closely resembling the architecture of actual human tissues. This advancement not only facilitates a more profound understanding of human biology and disease pathogenesis but also opens new avenues for drug testing and personalized medicine. By replicating the physiological context of human organs, organoids act as a more predictive model for human responses to therapeutic interventions, significantly minimizing the reliance on animal models and offering a more ethical and pertinent alternative for research.

Despite the enthusiasm for organoid technology, the transition from basic organoid models to complex biomimetic systems presents substantial challenges. Current organoid models, while impressive, do not fully replicate the multifaceted environment of the human gut, including intricate interactions with microbial communities, immune cells, and neural networks. Addressing these limitations demands innovative approaches to organoid engineering, including the integration of diverse cell types to resemble the gut’s immune and neural components (Bar-Ephraim et al., 2019; Park et al., 2020), and the application of advanced fabrication techniques such as Microfluidic to develop more sophisticated organoid architectures (Brassard and Lutolf, 2019). Furthermore, the development of organoid models that incorporate human microbiota presents promising pathways to investigate the microbiome’s role in health and disease (Rooks and Garrett, 2016; Hill and Spence, 2017). This review aims to highlight the latest technological advancements in constructing complex intestinal organoid systems, as well as to discuss the significance of these advancements for disease modeling and drug discovery.

## 2 Gut organoids: composition and characteristics

Intestinal organoids, complex structures derived from stem cells, encapsulate a diverse array of intestinal epithelial cell types (Takahashi et al., 2021). Included among these are Lgr5^+^ intestinal stem cells, Paneth cells (which provide niche support and antimicrobial defense), absorptive enterocytes, mucus-secreting goblet cells, and hormone-producing enteroendocrine cells (Sato et al., 2009; Takahashi et al., 2021). This cellular diversity is essential for replicating the functional and structural complexity of the intestinal epithelium, offering a dynamic model for investigating tissue homeostasis, disease mechanisms, and therapeutic interventions.

Adult stem cells (ASCs) are the primary source cells for constructing intestinal organoids. These intestinal stem cells retain characteristics of their original location in the gut, as well as genetic and epigenetic mutations of the host (Schreurs et al., 2019). Intestinal stem cells, isolated from hosts of any age, can be cultured *in vitro* within a 3D environment to construct intestinal organoids featuring host-specific genetics (Dotti et al., 2017; Howell et al., 2018). This breakthrough enables the development of organoids that closely replicate the genetic background of the original tissue. These organoid systems enable the investigation of genetic or epigenetic mutations and their impacts on biology, providing insights into the functioning and dysfunctions of the human gut. Another method for constructing intestinal organoids involves the directed differentiation of pluripotent stem cells (PSCs), including embryonic stem cells (ESCs) and human-induced pluripotent stem cells (iPSCs). PSC-derived intestinal organoids a complex, time-intensive, and highly specific developmental regimen. This process entails the addition of specific growth factors at precise dosages and timings to guide PSCs towards forming 3D intestinal epithelial structures (McCracken et al., 2014). However, PSC-derived organoids encounter genomic instability issues, attributed to exposure to reprogramming factors and an inherently immature phenotype (Günther et al., 2022). This instability poses significant challenges in accurately replicating the genetic and functional characteristics of the target organ.

While basic organoid models have significantly enhanced our understanding of intestinal biology and disease, their application is limited by a lack of complexity, and they fail to fully replicate the intricate microenvironment of the gut (Günther et al., 2022). This limitation highlights the need to develop these models into more complex organoid systems that include additional components such as immune, neural, and vascular cells. Enhancing the complexity of organoids will bridge the gap between *in vitro* models and actual human physiology, unlocking new possibilities in drug discovery, personalized medicine, and the understanding of human biology at an unprecedented level.

## 3 Design and construction of complex gut organoids

**FIGURE 1:**
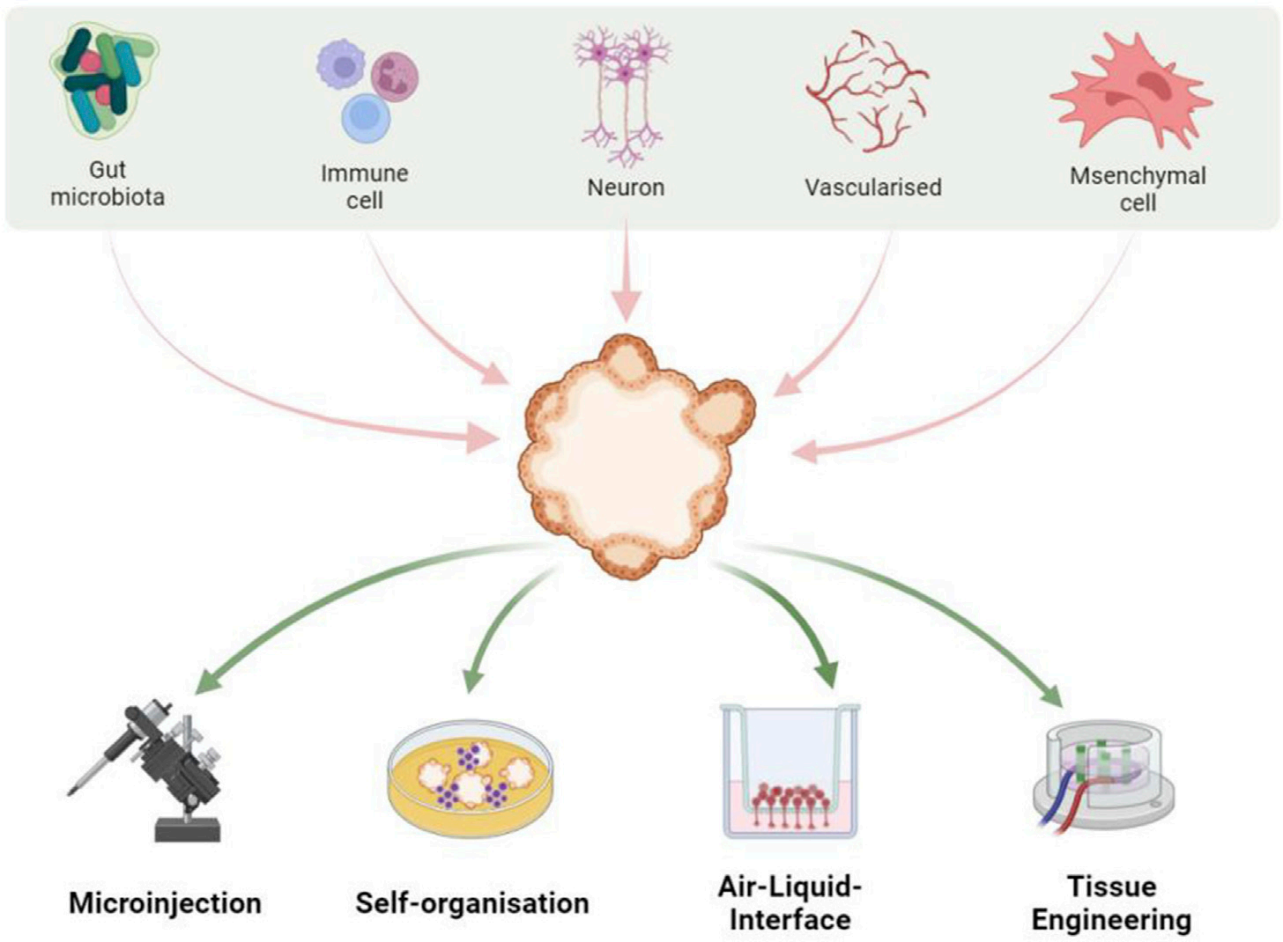
Constructing gut organoid platforms for complex systems through various approaches.

The gut comprises an intricately complex system, where the functionality of gut epithelial cells is influenced not only by dietary habits but also significantly by the interplay among immune cells, neural cells, and vascular endothelial cells (Bar-Ephraim et al., 2019; Liu et al., 2021; Lei et al., 2022; Wiggins et al., 2023). Furthermore, the gut microbiota is element in orchestrating gut functionality, highlighting its critical role within this ecosystem (Fassarella et al., 2021). Current gut organoid models, however, do not accurately reflect the multifaceted communications among these diverse cell types, limiting their utility in elucidating disease pathogenesis and in screening therapeutic agents. This indicates an urgent need for the development of advanced gut organoid platforms that can mimic the gut microenvironment with high fidelity, thus paving the way for more comprehensive insights into intestinal health and disease (Figure 1).

### 3.1 The microbe-intestinal organoids Co-culture systems

The gut microbiota plays a crucial role in health and disease, influencing a range of physiological processes, immune responses, and the efficacy and toxicity of drugs. Interactions between gut microbes and the host significantly affect the development and progression of diseases, such as intestinal disorders, metabolic syndromes, and infections (Fassarella et al., 2021). Simple intestinal organoids, primarily composed of epithelial cell types, lack the complexity of the *in vivo* gut environment, notably the absence of gut microbiota. This limitation restricts their utility in fully understanding host-microbe interactions and their impact on the gut’s physiological and pathological states.

Integrating the gut microbiome with intestinal organoids in co-culture systems marks a significant advancement in gut research. These models have played a pivotal role in elucidating the mechanisms through which microbes influence gut health, disease, and drug metabolism. In a groundbreaking development, researchers established a stem cell-derived air-liquid interface (ALI) culture system that successfully navigated the complexities of completing the Cryptosporidium parvum life cycle and enhanced genetic tractability *in vitro* (Wilke et al., 2019). This advance provides a readily available model that will facilitate future research on Cryptosporidium biology and its interactions with hosts. Furthermore, an innovative application of a co-culture model composed of human intestinal organoids and genotoxic pks + *E. coli* was introduced, marking the first demonstration of a direct association between exposure to pks + *E. coli* and the induction of specific oncogenic mutations linked to colorectal cancer in an *in vitro* environment. This method enabled a controlled examination of the mechanisms by which pks + *E. coli* induces DNA damage in intestinal epithelial cells, culminating in a distinctive mutational signature correlated with colorectal cancer (Pleguezuelos-Manzano et al., 2020). This research not only offers a new perspective on the microbiological factors involved in colorectal cancer but also establishes a potential foundation for future therapeutic strategies targeting such microbial interventions. Additionally, the co-culture of bat and human intestinal organoids with SARS-CoV-2 revealed robust viral replication in organoids of both species, indicating the human intestinal tract as a possible transmission route for SARS-CoV-2 and highlighting the significance of intestinal organoids in modeling enteric infections and exploring the origins of zoonotic diseases (Zhou et al., 2020). Another study introduced a novel methodology that involved the co-culture of intestinal organoids and the screening of microbiome-derived metabolites to identify the modulation of endoplasmic reticulum (ER) stress by gut bacterial metabolites. This approach successfully identified both inducers and suppressors of the ER stress response, highlighting the pivotal role of microbial metabolites in maintaining intestinal epithelial homeostasis and providing insight into the molecular dynamics of gut microbe-host interactions (Ke et al., 2021). Lastly, the use of co-culture techniques with intestinal organoids showed that outer membrane vesicles (OMVs) from engineered *E. coli* could facilitate the horizontal transfer of functional biomolecules, including Cre recombinase, between cell types *in vivo*. This technique reveals the extensive communication network facilitated by OMVs among the gut microbiota and various mammalian host cells, reaching remote organs beyond the gastrointestinal tract. This discovery emphasizes the profound impact of microbe-host interactions on mammalian physiology and the potential for disease modulation (Bittel et al., 2021).

From birth, the gut microbiota colonizes the host’s intestines, establishing a stable mutualistic relationship under healthy conditions. Several chronic diseases are linked to persistent alterations in the microbial composition. However, most epithelial co-culture studies span hours to days, inadequately simulating these chronic conditions (Puschhof et al., 2021). Studies on bacterial invasion in 2D cell lines typically require only a few hours (Rubinstein et al., 2013). In 3D organoids, the enclosed lumen allows stable co-culture of most gut microbes without inducing bacterial death or overgrowth. However, the necessity to passage organoids every 7–14 days limits stable co-culture beyond this period. A recent study by Pleguezuelos-Manzano et al. demonstrated the feasibility of constructing an organoid co-culture system by repeatedly injecting a single bacterial species over a 5-month period (Pleguezuelos-Manzano et al., 2020). Gut-on-a-chip systems have enabled the co-culture of complex bacterial communities or single species for durations ranging from several days to weeks (Kim et al., 2015; Jalili-Firoozinezhad et al., 2019). The latest generation of gut-on-a-chip systems can also support co-culture on hollow tubes for up to a month, accurately replicating the composition of intestinal cell types (Nikolaev et al., 2020). This technology allows for deep insights into the complex long-term relationships between microorganisms and host cells. Culturing the aerobic eukaryotic parasite Cryptosporidium for up to 4 weeks can be achieved in adult stem cell-derived organoids (Heo et al., 2018).

Despite these innovations, several gaps and limitations remain that need addressing to fully exploit the potential of these co-culture systems. A primary gap in the current research is the limited representation of the full gut microbiome diversity and its complex interactions within co-culture systems. Most studies focus on single-species or limited consortia of microbes, failing to capture the full complexity of microbial communities in the human gut. In contrast, simplistic models of individual microbial effects can easily exaggerate the impact of specific gut microbiota, overemphasizing the study’s benefits and leading to inaccurate results (Walter et al., 2020). Additionally, the dynamic interactions between the microbiome and the host’s immune system are inadequately modeled, limiting our understanding of their contribution to health and disease. Maintaining a long-term co-culture system of gut microbiota and intestinal organoids is a critical challenge that needs to be addressed. This will help us to better understand the contribution of gut microbiota to intestinal health and facilitate the discovery of new drugs for treating chronic diseases, since the gut microbiota also influences drug metabolism, thereby affecting drug efficacy (Geller et al., 2017; Zimmermann et al., 2019).

Looking ahead, the field must strive to overcome these challenges with targeted research and innovation. Enhancing the diversity of microbial communities incorporated into co-culture systems is crucial to accurately reflect the complexity of the human gut microbiome. This involves including a wider range of bacterial species and integrating other less-studied components of the gut microbiome, such as fungi and viruses. Incorporating immune cells into these models is critical, enabling a more comprehensive understanding of the microbiome’s interaction with the host’s immune system. Improving the physiological relevance of these models is paramount and could be achieved by developing advanced microfluidic systems designed to more accurately mimic the dynamic conditions of the gut. Microfluidics combined with organ-on-a-chip technology, through the precise regulation of oxygen concentration, nutrient components, and other conditions, may be a potential strategy to overcome the challenges of maintaining long-term stability in gut microbiota and organoid co-culture systems. Establishing standardized protocols for creating and maintaining co-culture systems is crucial for enhancing the reproducibility and comparability of research findings across the field. By addressing these future research directions, we can fully realize the potential of co-culture systems to illuminate the complexities of gut microbiome-host interactions and their implications for health and disease.

### 3.2 Simulating the intestinal microenvironment with multicellular organoids

Creating multicellular organoid models to simulate the intestinal tissue microenvironment is crucial for accurately replicating the complex interactions within the gut. These models incorporate various cell types, including immune cells, neural cells, vascular cells, and stromal cells, each contributing uniquely to GI physiology. This approach facilitates a deeper understanding of the gut’s cellular ecosystem, providing insights into disease mechanisms, therapeutic responses, and tissue regeneration processes.

#### 3.2.1 Immune cell and intestinal organoid co-culture systems

The advent of co-culture systems that combine immune cells with intestinal organoids has heralded a new era in gut immunology research, providing unparalleled insights into the complex interplay between the gut epithelium and the immune system. These innovative models have played a key role in elucidating the mechanisms that underlie mucosal homeostasis, immune responses to pathogens, and the restoration of epithelial integrity following injury.

In Dijkstra et al., a novel technique was employed to cultivate tumor-reactive T cells through the co-culture of peripheral blood lymphocytes with autologous tumor organoids. This method significantly enriched tumor-specific T cells in the blood of patients with mismatch repair-deficient colorectal cancer and non-small cell lung cancer, opening up a new avenue for the personalized assessment of anti-cancer immune responses (Dijkstra et al., 2018). Furthermore, Nozaki et al. developed a novel co-culture system that integrates intraepithelial lymphocytes (IELs) with intestinal epithelial cells (IECs), organized into three-dimensional organoids. This development successfully preserves and examines the motility of IELs *in vitro*, facilitating the exploration of dynamic interactions between IELs and IECs. This innovative approach underscores the significance of intestinal organoids as an indispensable resource for investigating the intricate behaviors and functions of immune cells within the gut epithelium (Nozaki et al., 2016). Additionally, the same team developed a pioneering co-culture framework combining IELs with IECs cultivated as three-dimensional organoids. This strategy enabled the efficient expansion and detailed analysis of IEL motility, demonstrating the potential of intestinal organoids as a powerful tool for elucidating the complex interactions and functionalities of immune cells within the gut epithelium (Neal et al., 2018).

The gut immune system is extensive, widely distributed across the lamina propria, epithelial layer, and lymphoid follicles. These structures are collectively referred to as gut-associated lymphoid tissue (GALT) (Mörbe et al., 2021). Although significant progress has been made in co-culturing intestinal organoids with various immune cells, these models still do not fully replicate the complex immune functions of the human gut. Carine Bouffi et al. developed the first intestinal organoids with a functional immune system (Bouffi et al., 2023), Their model features GALT-like structures and replicates the increase in M cells, activation of immune cells, and production of IgA following bacterial infection. This provides a powerful tool for studying gut development and intestinal homeostasis. However, the complex and time-consuming construction of this model may limit its current applicability for high-throughput drug sensitivity testing.

However, numerous limitations remain in the current methodologies. A major challenge involves replicating the complete immune cell milieu found *in vivo*, as most studies are focused on interactions with a limited subset of immune cells. This limits the understanding of comprehensive immune responses within the gut environment. Additionally, maintaining the physiological relevance of these co-cultures over extended periods, to study chronic immune responses or long-term effects of interventions, is technically challenging.

Future research should focus on integrating a broader spectrum of immune cell types within co-culture systems to more closely mimic *in vivo* conditions. Developing methodologies that sustain these co-cultures for extended durations is crucial for studying chronic conditions, such as inflammatory bowel disease. Incorporating microfluidic and organ-on-a-chip technologies represents another promising direction to more accurately replicate the dynamic interactions and gradients present in the gut environment. By addressing these gaps, we can significantly enhance the utility of co-cultured intestinal organoids as models for understanding gut immunology and developing targeted therapies for gastrointestinal diseases.

#### 3.2.2 Integration of enteric nervous system and intestinal organoids

The enteric nervous system (ENS) plays a crucial role in regulating gastrointestinal functions, including motility, secretion, blood flow, and mucosal immune functions (Engel et al., 2011; Jarret et al., 2020; Lei et al., 2022). Recent advancements in co-culturing ENS components with intestinal organoids have significantly deepened our understanding of gut physiology and neuroepithelial interactions. These innovative co-culture systems replicate the intricate cellular dynamics of the gastrointestinal tract, offering a model that is more physiologically relevant than traditional methods.

A groundbreaking method was developed for creating human pluripotent stem cell-derived intestinal tissues, incorporating a functional enteric nervous system (ENS) via symbiotic co-culture of derived neural crest cells with human intestinal organoids. This innovative technique not only replicated the ENS development within intestinal tissues in a controlled environment, but it also established an model for investigating ENS-associated disorders and exploring potential regenerative treatments (Workman et al., 2016). Furthermore, researchers introduced colonic organoids engineered from human embryonic stem cells, uniquely incorporating both enteric nerves and blood vessels, using a novel three-dimensional co-culture approach. This breakthrough offers a more comprehensive model for studying colon diseases by incorporating vital tissue components essential to organ function, significantly advancing regenerative medicine research for colon disease and beyond (Park et al., 2020). Additionally, a novel methodology created functional human gastrointestinal organoids from the three primary germ layers: enteric neuroglial, mesenchymal, and epithelial precursors, each independently derived from pluripotent stem cells. This process yielded complex human gastric organoids with three germ layers, featuring differentiated glands encased in smooth muscle layers and functional enteric neurons. This advancement in tissue engineering opens a new avenue for investigating human gastrointestinal development and the mechanisms behind various diseases (Eicher et al., 2022).

The brain controls intestinal peristalsis and secretion of digestive fluids through the autonomic nervous system (sympathetic and parasympathetic nerves), while the gut provides feedback and influences brain function. This bidirectional interaction between the gut and the central nervous system is known as the gut-brain axis (GBA) (Wu et al., 2023). Advances in organoid technology have opened up new approaches for understanding the gut-brain axis (GBA). Martin Trapecar et al. developed an integrated microfluidic system for studying the gut-liver-brain axis. This system connects the microenvironments of the intestine and liver with brain organoids created from human pluripotent stem cells. In this process, CD4^+^ T cells were added to the culture medium to simulate the characteristics of Parkinson’s disease patients (Trapecar et al., 2021). Research on the gut-brain axis contributes to a better understanding of gastrointestinal dysfunctions and neurological disorders.

While co-culturing ENS cells with intestinal organoids represents a significant advance in modeling the human gut’s complexity, it is not without challenges. A primary limitation involves replicating the complete neural cell milieu of the ENS within organoids. Most studies focus on incorporating a limited subset of neural cells, failing to fully capture the diversity and complexity of neuronal and glial cell types present in the ENS. Additionally, establishing the long-term viability and functionality of co-cultured neural cells within intestinal organoids poses significant technical challenges. Considering the functional connections between various neurons in the central nervous system and the intestinal epithelium is crucial. This will help us understand the mutual influence of gut physiology on the nervous system.

Future research should aim to address these gaps by developing methods that incorporate a broader spectrum of ENS cell types into organoid models, including neurons and glia, to better reflect the cellular diversity of the native ENS. Efforts should also focus on enhancing the physiological relevance of these models by integrating microfluidic and organ-on-a-chip technologies, to recreate the dynamic physical and biochemical environment of the gut. Furthermore, applying advanced imaging and sequencing techniques will be crucial for characterizing the complex interactions between neural cells and intestinal epithelial cells within co-cultures, providing insights into the mechanisms influencing gut physiology and pathology. By overcoming these challenges, co-culturing neural cells with intestinal organoids has the potential to significantly advance our understanding of gut-brain axis interactions, neurogastrointestinal disease pathogenesis, and the development of novel therapeutic strategies targeting the ENS.

#### 3.2.3 Vascularization in intestinal organoids

The introduction of vascularized intestinal organoids represents a significant milestone in the evolution of *in vitro* models, aiming to more accurately mimic the intricate microenvironment of the human intestine. Traditional organoid models, though providing valuable insights into intestinal biology, are limited by their lack of vascular structures, crucial for nutrient delivery, waste removal, and the mimicry of *in vivo* physiological conditions (Rafii et al., 2016).

In the study by Holloway et al., a novel approach was devised to differentiate and maintain an endogenous population of endothelial cells (ECs) within human pluripotent stem cell-derived intestinal organoids (HIOs) through the modification of culture conditions (Holloway et al., 2020). This method utilizes single-cell RNA sequencing to identify and expand the vascular endothelium within the organoids, yielding a more physiologically relevant model including critical vascular components. Thus, the study significantly enhances the complexity and potential utility of HIOs for research in human intestinal development, disease modeling, and regenerative medicine. Additionally, a groundbreaking three-dimensional co-culture method was utilized to engineer colonic organoids from human embryonic stem cells, featuring both enteric nerves and blood vessels (Park et al., 2020). This method tackled the challenge of incorporating vital tissue components, like nerves and blood vessels, into organoid models, enabling more thorough studies of colon diseases by incorporating critical physiological features. This progress not only expands the boundaries of research in colon diseases but also highlights the immense potential of intestinal organoids as a comprehensive model for dissecting human gastrointestinal physiology and pathology. Takebe et al. developed a generalized method for forming vascularized and complex organ buds from diverse tissues (including kidney, pancreas, intestine, heart, lung, and brain) through mesenchymal cell-driven condensation (Takebe et al., 2015). This approach combines pluripotent stem cell-derived tissue-specific progenitors or relevant tissue samples with endothelial and mesenchymal stem cells (MSCs), leveraging MSCs to initiate condensation based on substrate matrix stiffness. This technique not only highlights intestinal organoids’ application in modeling organogenesis and disease but also expands regenerative medicine’s potential by enabling the generation of functional, vascularized organ tissues *in vitro*. A microfluidic platform, IFlowPlate, is designed for culturing up to 128 independently perfused and vascularized colon organoids *in vitro*. This “open-well” platform, not requiring external pumping systems, enables tissue extraction for downstream analyses and *in vivo* transplantation (Rajasekar et al., 2020). It showed significant growth improvement in patient-derived colon organoids within a self-assembled vascular network under constant perfusion, compared to static conditions. Moreover, this development introduced a colon inflammation model with innate immune function, showcasing the platform’s utility in disease modeling and drug screening by offering a more physiologically relevant environment for organoid culture. This approach underscores the potential of incorporating vascularization into organoid systems to more closely mimic *in vivo* conditions, advancing the field of tissue engineering and regenerative medicine.

Despite these advancements, developing vascularized intestinal organoids presents several challenges. A primary limitation involves the incomplete replication of the native vascular architecture and its complex interactions with intestinal epithelial cells. While methods such as the gut organoid flow chip (GOFlowChip) and dynamic modulation of WNT signaling have been developed to incorporate vasculature into organoids, these techniques often yield vascular networks lacking the full functionality and structural complexity of *in vivo* systems. Compared to *in vivo* intestinal vasculature, a major drawback of vascularized organoids is their long-term static culture. Due to the physical limitations of passive diffusion of nutrients and oxygen, extensive cell death readily occurs in the core region. The developmental stage of organoids still lags behind normal adult tissues, resembling more of a fetal-like state, which also limits further vascular development. Additionally, the size and density of the vascular structures in organoids differ markedly from those *in vivo*, making them suitable only for short-term studies. Additionally, maintaining the long-term viability and functionality of these vascularized organoids *in vitro* is challenging, limiting their application in chronic disease modeling and drug testing. Future research should focus on enhancing the fidelity of engineered vascular networks within intestinal organoids, possibly through co-culturing with endothelial cells derived from pluripotent stem cells or incorporating microengineering techniques to more closely mimic the physical cues of the intestinal microenvironment. Further exploration of the signaling pathways and cell-cell interactions that govern vasculature development in the gut is also necessary. Addressing these gaps will not only enhance our understanding of intestinal biology but also propel the development of more effective therapies for gastrointestinal diseases.

#### 3.2.4 Mesenchymal and intestinal organoid co-culture

The interaction between interstitial cells and intestinal organoids in co-culture systems has emerged as a cornerstone in advancing our understanding of intestinal physiology and disease. These co-cultures have shed light on the complex interplay between epithelial cells and the immune system, underscoring the importance of such models in more accurately mimicking the intricate environment of the human gut compared to traditional cell culture methods.

The study by Jarde et al. utilized mesenchymal niche-derived neuregulin-1 (NRG1) to drive intestinal stem cell proliferation and regenerate damaged epithelium, revealing NRG1’s critical role in intestinal tissue regeneration by robustly stimulating proliferation in crypts and enhancing regeneration capacities (Jarde et al., 2020). This underscores the utility of intestinal organoids as a platform for studying stem cell-mediated tissue repair and regeneration mechanisms, showcasing intestinal organoids’ value in modeling cellular responses to specific growth factors in the gut microenvironment. This research used a combination of murine and human organoid models, mouse genetics, and high-throughput sequencing to identify TGFβ1 signaling as essential for epithelial regeneration post-injury (Chen et al., 2023). TGF-β1 was found to play a critical role in driving the intestinal epithelium into a fetal-like regenerative state, necessary and sufficient for this process. This discovery highlights the potential of pre-treating with TGF-β1 to enhance organoid engraftment into damaged colonic tissues, presenting a promising strategy for cellular therapy in intestinal regeneration.

The existing literature identifies gaps and limitations in current approaches. A primary challenge involves achieving an accurate replication of the diverse and dynamic stromal environment found *in vivo*, crucial for supporting intestinal epithelial function, regulating stem cell niche signals, and mediating immune responses. Most studies, including those described by Wörsdörfer et al. (Worsdorfer et al., 2020) and others, have focused on co-culturing specific types of stromal cells, including mesenchymal stem cells or fibroblasts, with organoids. While these efforts have elucidated certain aspects of epithelial-stromal interactions, they often fail to capture fully the complexity of stromal cell types and their interactions within the gut. Additionally, maintaining the long-term viability and functionality of co-cultured stromal and epithelial cells represents a significant technical challenge, as current models often fail to sustain multicellular interactions over extended periods. This limitation restricts the study of chronic diseases and the long-term effects of therapeutic interventions within these models.

### 3.3 Tissue engineering techniques in constructing intestinal organoids

Recent advancements in tissue engineering, utilizing microfluidics, organ-on-a-chip, and 3D printing technologies, have significantly advanced the development of complex intestinal organoid systems. These methods aim to incorporate dynamic cultivation models and introduce specific organizational morphologies, thereby offering innovative platforms for disease modeling, drug testing, and regenerative medicine (Günther et al., 2022). A comparative summary of different organoid model systems is provided in Table 1 (Daly et al., 2021; Wang et al., 2024).

**TABLE 1:** Summary of different organoid model systems.

Model	Advantages	Limitations
2D cell	• Simple to operate, does not require complex culture conditions • Facilitates high-throughput screening of drugs • Suitable for straightforward preclinical studies • Low cost	• Primarily consists of genetically modified cell lines, with significant differences from human tissues • Does not capture the interactions between multiple cell types within an organ • Does not replicate the dynamic physiological conditions between organs • Challenging to sustain long-term culture to preserve tissue function
Intestinal Organoids	• Can simulate the main characteristics of human tissues with fidelity and include complex cellular components • Maintains cellular genotype and phenotype over the long term • Enables personalized drug screening	• Does not incorporate physical signals (shear stress, mechanical tension, gradients, etc.) • Does not include a complex tissue microenvironment • Depends on animal-derived culture reagents • Has a low success rate in constructing patient-derived organoids
Microfluidic organ-on-a-chip	• Integrates complex microenvironment components with physiological functions • Enables real-time monitoring and *in situ* observation • Helps bridge the gap between *in vitro* and *in vivo* experimental results • Serves as an alternative to animal models for drug testing and disease modeling	• Necessitates sophisticated instrumentation • Challenging to culture multiple types of organoids sequentially under consistent conditions for long-term studies • Necessitates the development of novel materials and dimensions to enhance the accuracy of drug sensitivity testing
3D Bioprinting	• Rapid prototyping • Highly customizable • Enables the creation of heterogeneous structures using multi-head bioprinters • Allows for the creation of structures with high spatial precision	• Exhibits lower precision and cannot accurately replicate the complexity of tissues • The printing speed of large and complex structures is slow, which affects cell viability • The produced models lack uniformity and reproducibility • High cost

#### 3.3.1 Microfluidic organ-on-a-chip

Gut chip models have demonstrated significant advantages in simulating interactions between the gut microbiota and human cells, offering new insights into intestinal physiology and pathophysiology. These models use microfluidic technology to accurately simulate key physiological stimuli in the gut, such as fluid flow, mechanical forces, and oxygen gradients, thereby enhancing the long-term co-culture of microbiota and human cells (Brassard and Lutolf, 2019; Fu et al., 2020). Microfluidic organ-on-a-chip models of the human intestine, as reviewed by Bein et al., offer dynamic environments that mimic the physiological conditions of the gut, including fluid flow and mechanical forces (Bein et al., 2018). An innovative intestine-on-a-chip system was developed, capable of culturing a complex human gut microbiome under anaerobic conditions directly in contact with the living human intestinal epithelium. This system, featuring integrated microscale oxygen sensors and a custom-engineered anaerobic chamber, enabled the establishment of physiologically relevant oxygen gradients. This advancement allows for extended co-culture of aerobic and anaerobic human commensal gut microbes with human intestinal cells, maintaining microbial diversity and supporting studies of host-microbiome interactions in health and disease, highlighting the organoid’s significant value in simulating the human gut environment for research and therapeutic development (Jalili-Firoozinezhad et al., 2019). A novel method for creating homeostatic mini-intestines through scaffold-guided organoid morphogenesis was developed, leveraging tissue engineering and cells’ intrinsic self-organization properties. This method induced intestinal stem cells to form tubular epithelia that mimic the *in vivo* spatial arrangement of crypt- and villus-like domains, providing an accessible lumen and perfusion capability. This advancement enables the long-term culture of intestinal tissues, supports colonization with microorganisms for modeling host-microorganism interactions, and includes rare specialized cell types not commonly found in conventional organoids. This method highlights the utility of intestinal organoids as a versatile tool for studying intestinal biology, disease mechanisms, and host-pathogen interactions in a more physiologically relevant context (Nikolaev et al., 2020). These platforms enable the study of intestinal physiology and disease in a controlled setting, allowing for the investigation of cellular interactions, barrier function, and response to pathogens. Despite their potential, the complexity of microfluidic systems and challenges associated with integrating multiple cell types and replicating the full spectrum of gut microenvironments can limit them. Choosing biomaterials and fabricating organ chips is crucial for successfully mimicking complex organ systems. Recent research highlights the application of various biomaterials and manufacturing technologies, such as polydimethylsiloxane (PDMS), detailing their advantages and disadvantages in constructing gut chip models (Cao et al., 2023). While advancements in these technologies have enhanced the feasibility and practicality of organ chip models, cost and technical challenges remain significant obstacles.

#### 3.3.2 3D Bioprinting

Bioprinting employs computer-aided transfer processes to pattern and assemble biological and non-biological materials in predefined 2D or 3D organizations to produce bioengineered structures (Moroni et al., 2018). Brassard et al. introduced a bioprinting concept using organoid-forming stem cells as building blocks, capable of being deposited directly into extracellular matrices conducive to spontaneous self-organization (Brassard et al., 2021). By controlling the geometry and cellular density, they generated centimetre-scale tissues featuring self-organized elements like lumens, branched vasculature, and tubular intestinal epithelia with in vivo-like crypts and villus domains. Spheroid-based bioprinting technology can accurately construct cell-dense 3D structures or organoid-containing structures (Moldovan et al., 2017). This approach, termed Bioprinting-Assisted Tissue Emergence (BATE), illustrates how biofabrication and organoid technology can merge to control tissue self-organization from millimetre to centimetre scales, opening new avenues for drug discovery, diagnostics, and regenerative medicine.

## 4 Intestinal organoid systems for disease modelling and drug discovery

The construction of complex intestinal organ systems has emerged as a pivotal area of research in gastrointestinal physiology, disease modeling, and drug screening. Advances in this field have been driven by the development of gut-on-a-chip models and organoids derived from human pluripotent stem cells, each offering unique insights into the intricate biology of the human intestine.

Krüger et al. utilized human pluripotent stem cell-derived intestinal organoids (PSC-HIOs) to model SARS-CoV-2 infection, demonstrating the virus’s ability to infect and replicate within these organoids (Krüger et al., 2021). This study assessed the efficacy of potential treatments, finding remdesivir and EK1 significantly inhibited viral replication within PSC-HIOs, while famotidine did not show a similar effect. This method highlights PSC-HIOs’ value as a relevant and sophisticated model for understanding SARS-CoV-2 pathogenesis in the gut and screening for effective treatments, marking a significant advancement in COVID-19 research and therapeutic strategy development for gastrointestinal manifestations. The study by Günther et al. revealed IFNL upregulation in the ileal tissues of Crohn’s disease (CD) patients, promoting Paneth cell death via STAT1 signaling—a process replicated in mouse models (Günther et al., 2019). This research offers significant insights into CD’s pathogenesis, highlighting IFNL’s detrimental role in intestinal epithelium integrity and its therapeutic target potential for CD management, emphasizing the utility of intestinal organoid models for disease modeling and drug screening. Han et al. developed human pluripotent stem cell-derived lung and colonic organoids (hPSC-LOs and hPSC-COs) as *in vitro* platforms for high-throughput drug screening against SARS-CoV-2 (Han et al., 2020). They showed that these organoids are susceptible to SARS-CoV-2 infection and identified several FDA-approved drugs, including imatinib, mycophenolic acid (MPA), and quinacrine dihydrochloride (QNHC), that significantly inhibit SARS-CoV-2 infection. This study underscores the utility of hPSC-derived organoids as versatile and physiologically relevant models for studying viral infections and screening for therapeutic agents, offering a powerful tool for rapid response to emerging infectious diseases. Saito et al. used clonal analysis of human colon organoids derived from ulcerative colitis (UC) patients to reveal a distinct pattern of somatic mutagenesis in the inflamed UC epithelium, particularly in genes related to IL-17 signaling (Nanki et al., 2019). This approach demonstrated the organoids’ utility in uncovering genetic alterations associated with chronic inflammation and their contribution to the pathogenesis of UC, emphasizing the value of intestinal organoids in genetic and pathophysiological studies of inflammatory bowel diseases.

The utilization of intestinal organoid technology for disease modeling and drug screening represents a promising avenue for advancing our understanding of gastrointestinal disorders and the development of novel therapeutics. Yuxuan Zhu et al. have developed a high-throughput drug screening organoid platform containing 304 microchannel wells, which is capable of culturing 304 identical organoids simultaneously on a chip. This platform enables the evaluation of multiple drugs or different concentration gradients of the same drug, significantly shortening the experimental cycle (Zhu et al., 2024). A recent study introduced a PDMS-free multi-organoid microfluidic platform that can accommodate up to six different organoids, thereby constructing a comprehensive microphysiological system (Skardal et al., 2020). This platform employs a 96-well non-adhesive round-bottom plate to culture liver, heart, lung, testis, colon, and brain organoids, which can then be freely combined in a microfluidic platform to construct a six-tissue microphysiological system. Using this system, they successfully demonstrated that the liver organoids could metabolize capecitabine and cyclophosphamide into their activated toxic forms, thereby producing downstream drug toxicity in other organoids. In addition, a study reported utilizing a high-throughput microwell array combined with live/dead fluorescent staining for the culture of tumor organoids, conducting both single and combination drug testing (Schuster et al., 2020). The platform integrates 200 microwell arrays, with the manual introduction of spheroids or organoids cultured *in vitro* into each microwell for drug testing. In addition to enhancing drug screening through the microscopic scale of microwell channels, integrating macroscopic biomimicry to construct inter-organ crosstalk on a high-throughput basis simulates physiological communication between organs *in vivo*. This approach aids in early drug screening and personalized therapy.

Despite considerable advancements, a key limitation is the inability of organoids to fully recapitulate the complex architecture and cellular diversity of the human intestine (Vargas-Valderrama et al., 2020; Múnera et al., 2023). While organoids can mimic certain aspects of the intestinal epithelium, they often lack critical components such as immune cells, neural cells, and the vasculature, which are significant in gut physiology and pathology. This shortfall affects the accuracy of disease models and the efficacy of drug screening processes (Koike et al., 2019). Another challenge is the variability in organoid generation and maintenance, leading to inconsistencies in experimental outcomes (Fleischer et al., 2020). Standardizing organoid culture conditions, such as the use of defined growth factors and substrates, is crucial for enhancing reproducibility across studies. Although intestinal organoids offer a more physiologically relevant model than traditional 2D cultures, technical challenges like automation and scaling limit their use in high-throughput drug screening (Wang and Jeon, 2022). Developing efficient methods to culture and assay organoids for high-throughput applications is essential for their broader use in drug discovery.

## 5 Challenges and future directions

### 5.1 Technical challenges and limitations

The development of complex intestinal organ systems, including gut-on-a-chip models and organoids, has revolutionized human gastrointestinal physiology and disease studies. However, simulating intestinal tissue *in vitro* presents significant challenges. These challenges include accounting for cellular heterogeneity, microbial interactions, precise mechanochemical extracellular matrix (ECM), biochemical and biophysical gradients, luminal flow and muscle peristaltic contractions, and vascular and lymphatic systems, all integrated into an accurate 3D tissue architecture (Cameron et al., 2023). Current co-culture models and bioengineering approaches are gradually integrating these cells, features, and functions with the intrinsic self-organization capability of intestinal organoids to develop gut microphysiological systems. However, a limitation is that intestinal organoids cannot fully represent adult intestinal epithelium, as their transcriptomic profile resembles that of fetal intestinal cells (Yu et al., 2021). Factors such as the intestinal epithelium and stem cell niche (Tetteh et al., 2016), various growth factor concentration gradients (Fre et al., 2005), oxygen gradients (Zheng et al., 2015), microbial gradients (Puschhof et al., 2021), mechanical gradients (Huizinga et al., 2014), and matrices supporting 3D culture of intestinal cells (Meran et al., 2017) cannot be accurately simulated *in vitro*. An important question to consider is what evidence supports the advantages of organoid systems over animal models, and to what extent organoid systems can replicate actual human physiological responses (Wang et al., 2024). To answer this question, it is necessary to standardize protocols for maintaining the physiological parameters of organoid systems. This includes identifying cell populations, developmental stages, and spatial distributions through transcriptomic profiling and visualization techniques, combining these data with patient biobank samples for real-time tracking and evaluation. Such standardization will greatly enhance the potential of organoid systems in high-throughput drug screening and personalized therapy.

### 5.2 Ethical issues and safety considerations

As with any emerging biotechnological approach, constructing complex intestinal organ systems raises ethical issues and safety considerations. The use of human stem cells, especially induced pluripotent stem cells (iPSCs), in creating intestinal organoids, introduces concerns about the source of these cells and the potential for creating genetically modified human tissues (Hamano et al., 2022). Questions also arise about the long-term implications of using organoids and gut-on-a-chip systems for personalized medicine, including genetic information privacy and security, and equitable access to these advanced therapeutic options (Rossi et al., 2018; Mollaki, 2021; de Jongh et al., 2022). Additionally, the potential for these systems to lead to more complex, possibly sentient, organ systems raises further ethical debates on the nature of consciousness and the rights of artificially created biological entities. Organoid research holds the potential for revolutionary advancements in disease development, drug screening, and biotherapy. Currently, several projects have already entered clinical translation and commercial applications. In this process, it is crucial to protect the privacy of organoid tissue donors and operate within ethical frameworks to maximize the utility and benefit of organoids. Before commercialization, it is essential to evaluate and address effective strategies to protect donor privacy. Whether organoids can be bought, sold, or exchanged, and how the value and commercial profits of commercialized organoids are distributed, should be thoroughly discussed among tissue donors, hospitals, research institutions, scientists, enterprises, and the relevant public.

### 5.3 Future research directions and potential applications

Intestinal organoid systems, due to their advantages in high-throughput *in vitro* culture, have gained widespread attention and intensive research as disease models for screening diagnostic markers, identifying therapeutic targets, and drug screening. Furthermore, as a transplantable cell system, organoid systems hold broad application potential in regenerative medicine and personalized medicine. Serious complications caused by gastrointestinal diseases such as inflammatory bowel disease (IBD) or short bowel syndrome (SBS) can be addressed with intestinal transplantation, which provides a new therapeutic option for these severe cases. Satoshi et al. successfully repaired the intestinal epithelium in UC model mice by transplanting epithelial organoids *in situ* into the colon of recipient mice via the rectum (Watanabe et al., 2022). Sato et al. discovered that transplanting ileal organoids into the colon of SBS rats induced the colon to develop small intestine-like characteristics (Sugimoto et al., 2021). However, transplant failure and the low availability of transplantable organs remain significant challenges. Additionally, post-transplant vascularization and neural integration of the graft are major obstacles to overcome. The development of intestinal tumor organoids enables the utilization of patient tumor samples to customize personalized treatment plans. By testing these organoids’ responses to various drugs, doctors can select the treatment regimen with the best therapeutic effect. A pioneering Phase I/II clinical trial employed an organoid biobank derived from patients with metastatic colorectal cancer and gastroesophageal cancer, demonstrating for the first time the value of patient-derived organoids in personalized medicine (Vlachogiannis et al., 2018). Yao et al. utilized 96 colorectal cancer organoids derived from 80 patients and found a strong correlation between the chemotherapy responses of patient-derived organoids and the actual responses of the corresponding patients. The accuracy rate was as high as 84.43%, with a sensitivity of 78.01% and a specificity of 91.97% (Yao et al., 2020). At present, multiple hospitals and research institutions are working on building such organoid biobanks to provide personalized services for patients. However, achieving personalized treatment requires robust genetic testing, comprehensive clinical data support, and close collaboration among medical teams.

## 6 Conclusion

Future research will likely focus on overcoming challenges by developing more sophisticated and integrated models to more accurately mimic the intestinal ecosystem. This includes better incorporating immune and nervous system components, as well as the microbiota, into intestinal organ systems. Additionally, navigating ethical and safety considerations will require the establishment of robust ethical guidelines and regulatory frameworks.

The potential applications of advanced intestinal organ systems in personalized medicine, drug discovery, and the understanding of human intestinal diseases are immense. By providing more physiologically relevant models, these systems have the potential to revolutionize our approach to diagnosing, treating, and preventing gastrointestinal diseases. Moreover, they provide a promising platform for studying the complex interactions between the host and its microbiota, crucial for developing novel therapeutic strategies targeting the gut microbiome.

In conclusion, while significant progress has been made in constructing complex intestinal organ systems, future research is crucial to address current challenges and unlock the full potential of these innovative models. Through interdisciplinary collaboration and technological innovation, the next-generation of intestinal organ systems will undoubtedly play a pivotal role in transforming gastrointestinal research and healthcare.
